# Environmental drivers of harbour porpoise fine-scale movements

**DOI:** 10.1007/s00227-018-3346-7

**Published:** 2018-04-30

**Authors:** Floris M. van Beest, Jonas Teilmann, Rune Dietz, Anders Galatius, Lonnie Mikkelsen, Dominique Stalder, Signe Sveegaard, Jacob Nabe-Nielsen

**Affiliations:** Department of Bioscience, Aarhus University, Frederiksborgvej 399, 4000 Roskilde, Denmark

## Abstract

Quantifying intraspecific variation in movement behaviour of marine predators and the underlying environmental drivers is important to inform conservation management of protected species. Here, we provide the first empirical data on fine-scale movements of free-ranging harbour porpoises (*Phocoena phocoena*) in their natural habitat. Data were obtained from six individuals, tagged in two areas of the Danish North Sea, that were equipped with Global Positioning System (GPS) and dive recorder units (V-tags). We used multi-model inference and model averaging to evaluate the relative importance of various static and dynamic environmental conditions on the movement characteristics: speed, turning angle, dive duration, dive depth, dive wiggliness (a proxy for prey chasing behaviour), and post-dive duration. Despite substantial individual differences in horizontal and vertical movement patterns, we found that all the tracked porpoises responded similar to variation in environmental conditions and displayed movements that indicate a higher likelihood of foraging behaviour in shallower and more saline waters. Our study contributes to the identification of important feeding areas for porpoises and can be used to improve existing movement-based simulation models that aim to assess the impact of anthropogenic disturbance on harbour porpoise populations.

**Electronic supplementary material:**

The online version of this article (10.1007/s00227-018-3346-7) contains supplementary material, which is available to authorized users.

## Introduction

The field of movement ecology is rapidly maturing due to continuous advances in biotelemetry (Rutz and Hays 2009; Wilmers et al. 2015) as well as the development of conceptual frameworks that aim to unify research in animal movement (Nathan et al. 2008; Allen and Singh 2016). An important component of movement ecology is to quantify the influence of environmental conditions on animal space use (Signer and Ovaskainen 2017). Although anthropogenic disturbance, predator avoidance, and social interactions are known to influence behaviour and space use patterns of marine species (e.g., Brakes and Dall 2016), movement behaviour of cetaceans is thought to be most strongly influenced by foraging on patchy prey (Palacios et al. 2013; Wisniewska et al. 2016). Quantifying predator–prey relationships at fine spatiotemporal scales in marine systems is, however, extremely challenging and only few studies have attempted to do so using coarse spatial scales (Sveegaard et al. 2012; Benoit-Bird et al. 2013). Instead, most studies rely on static (e.g., bathymetry and distance to coast) and dynamic abiotic variables (e.g., sea-surface temperature and salinity) to explain variation in marine predator movement behaviour to indirectly identify the spatiotemporal distribution of potentially important feeding areas (Johnston et al. 2005; Abascal et al. 2011; Sousa et al. 2016). Indeed, for marine predators, low speed and convoluted movements combined with longer and deeper dives typically indicate foraging behaviour, while fast and linear movements coinciding with shallow dives are considered travelling behaviour (Towner et al. 2016; Leos-Barajas et al. 2017). Identifying feeding habitat based on fine-scale movement behaviour is essential for the successful conservation of cetaceans that inhabit coastal shelf waters, as it can highlight areas of potential conflict with current or planned anthropogenic activities (Cooke 2008; Brakes and Dall 2016; Hays et al. 2016).

The harbour porpoise (*Phocoena phocoena*) is a small marine predator and among the most common cetacean species within European coastal shelf waters (Hammond et al. 2013). Nonetheless, the species has a high conservation status in the European Union (EU) as it is listed in Annexes II and IV of the EU Habitats Directive (EU 1992). To assist in management and conservation, research on habitat use and movement behaviour of harbour porpoises has grown markedly over the past years, exploiting a range of data collection methods that differ in spatiotemporal resolution and precision. At a rather coarse resolution, ARGOS satellite tags are frequently used to obtain location data to assess habitat use, home range size, and large-scale movement patterns (Johnston et al. 2005; Sveegaard et al. 2011; Linnenschmidt et al. 2013). The advantages of ARGOS tags are that location data can be collected over long time periods (months to years), yet the number of positional estimates acquired is often sparse with relatively high location error ranging from tens of meters to kilometres (Vincent et al. 2002). This feature limits the use of ARGOS data in robust assessments of the impact of dynamic abiotic conditions on fine-scale movements of individual animals. At an extremely fine resolution, digital multisensory tags that record sound, acceleration, and dive depth have recently provided extremely detailed observations of vertical movements and foraging behaviour of free-ranging porpoises (Wisniewska et al. 2016). However, lack of location data and short sampling duration of suction cup tags (< 2 days) limits examination of porpoise movements as a function of environmental variation and the identification of important foraging areas.

Our objectives were to provide a detailed description of fine-scale movement parameters of free-ranging harbour porpoises residing in the Danish part of the North Sea and to relate variation in movement behaviour to a range of static and dynamic environmental conditions. Some evidence exists that the availability and distribution of important porpoise prey, such as cod (*Gadus morhua*), herring (*Clupea harengus*), and sprat (*Sprattus sprattus*) (Sveegaard et al. 2012; Andreasen et al. 2017), are positively correlated with salinity and temperature in this area (Hedger et al. 2004; Akimova et al. 2016). We, therefore, expect these dynamic abiotic variables in particular to be important drivers of porpoise fine-scale movement behaviour.

## Materials and methods

### Study area

**Fig. 1:**
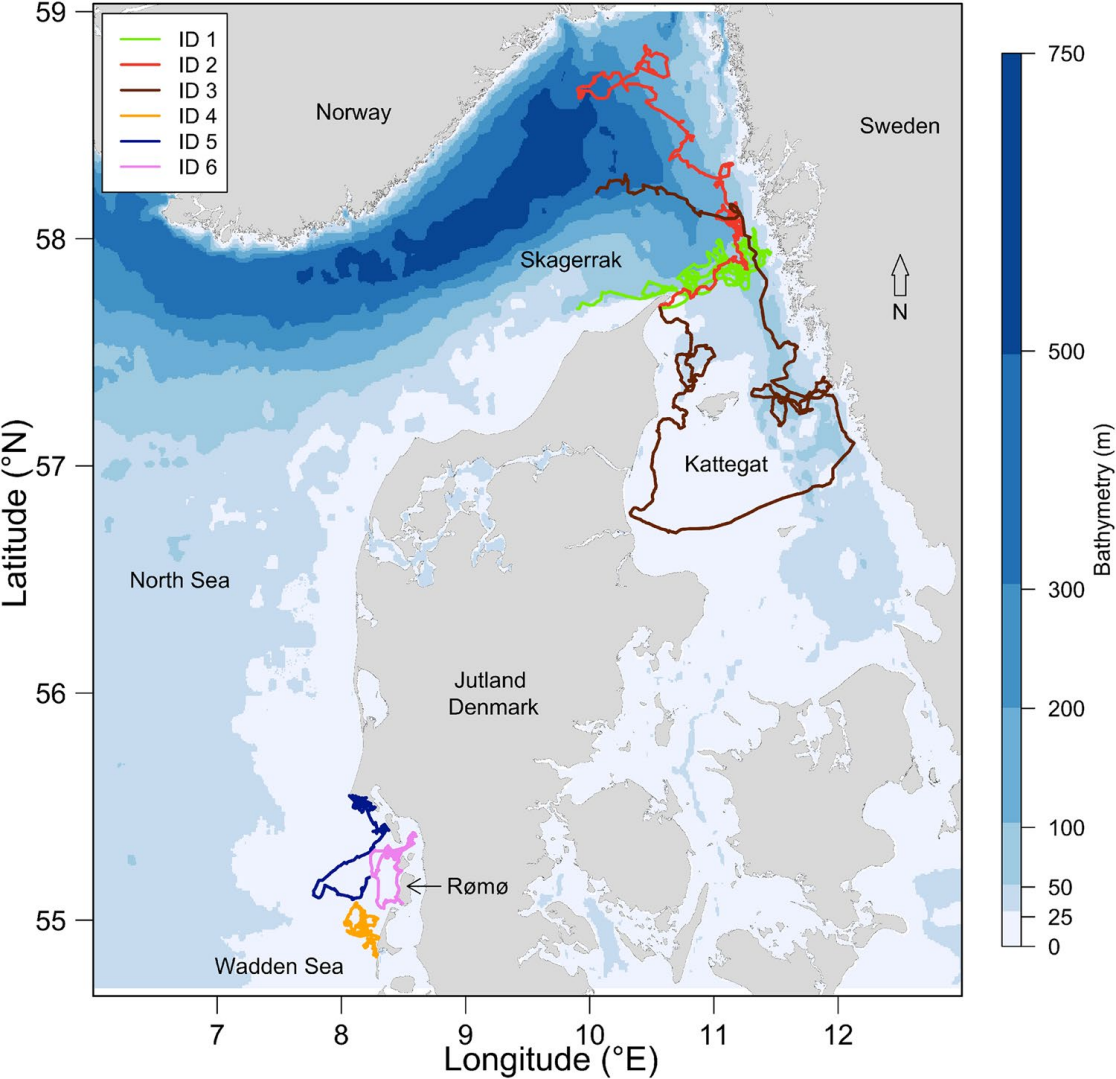
Map of the study area (including Kattegat, Skagerrak, North Sea, and Wadden Sea areas) showing bathymetry (m) of the region and movement trajectories of six harbour porpoises tagged with Fastloc GPS units as part of the V-tag

The study was conducted in the Danish parts of the Kattegat, Skagerrak and Wadden Sea (Fig. 1). The Skagerrak is a strait running between the southeast coast of Norway, the southwest coast of Sweden, and the Jutland peninsula in northern Denmark, connecting the North Sea and Kattegat. Skagerrak is ca. 240 km long and between 40 and 80 km wide and covers a total area of ca. 15,000 km^2^. The coastal parts of Skagerrak have shallower water depths (< 50 m), but most of Skagerrak consists of the Norwegian Trench that deepens down to 700 m. Sea-surface salinity levels in the area vary across seasons, but are typically lowest in Kattegat and increase towards Skagerrak and the North Sea (Supplementary Figure S1). Sandy bottom occurs in the shallow areas, while mud dominates the deeper areas of Skagerrak. The Wadden Sea in the south-eastern part of the North Sea has a total length of ca. 500 km and a total area of ca. 10,000 km^2^. It is a shallow body of water (< 25 m depth), large parts being intertidal zones with tidal flats and wetlands and is a recognized UNESCO world heritage site of international importance. Sea-surface salinity in the Wadden Sea area is more constant than in Skagerrak and varies little between seasons (Supplementary Figure S1). Harbour porpoises are present year round throughout both study areas (Sveegaard et al. 2011; Hammond et al. 2013; Gilles et al. 2016) and are considered to belong to the same genetically distinct population (Wiemann et al. 2010).

### Capture and tagging procedures

A total of six harbour porpoises were live caught and tagged between June 2014 and September 2016 (Table 1). Three porpoises were caught incidentally in pound nets around the northern tip of the Jutland peninsula (in Skagerrak) and three porpoises were caught actively in the Wadden Sea close to the Danish island Rømø.

**Table 1:** Overview of individual-, capture/tagging- and movement-related information for each of the six harbour porpoises equipped with a V-tag during this study

	Harbour porpoise ID					
	ID 1	ID 2	ID 3	ID 4	ID 5	ID 6
Sex	Female	Female	Male	Male	Male	Male
Standard length (cm)	138	139	134	120	140	130
Tagging area	Skagerrak	Skagerrak	Skagerrak	Wadden Sea	Wadden Sea	Wadden Sea
ARGOS no.	2015-149159	2015-149160	2015-149162	2014-138067	2016-149166	2016-149167
V-tag version	2	2	3	2	2	2
Location data available	Yes	Yes	Yes	Yes	Yes	Yes
Dive data available	Yes	Yes	Yes	Yes	Yes	No
Tagging date (d/m/y)	02/11/2015	02/11/2015	20/11/2015	03/06/2014	19/09/2016	19/09/2016
No. of days tagged	11	9	11	7	7	12
Total no. of GPS locations acquired	919	1138	1210	1353	594	1312
Average no. of GPS locations acquired h^−1^	3.8	5.7	4.7	9.6	3.7	4.8
Total no. of dives recorded (> 2 m, > 10 s)	9160	9674	15623	4575	8297	−
Average no. of dives h^−1^	38.2	48.6	53.1	32.4	52.2	−

The V-tag is a custom-made high-density closed cell foam package containing GPS, TDR, VHF, and ARGOS units. The V-tag version 2 had a weight of 150 g and version 3 was 135 g. Note that ID 6 did not have a functioning TDR (dive recorder) unit

Pound nets are used in near-shore commercial fisheries in the inner Danish waters and consist of fixed wooden poles where a lead net ends in a trap ca. 1 km from shore. The net trap typically measures 10–30 m in diameter and 3–15 m in depth and consists of a bag that opens at the surface with a mesh size of 2 × 2 cm. Pound nets pose no threat of drowning to the porpoises as they can breathe at the surface and swim freely while entrapped. Fishermen that encountered a porpoise in their pound nets contacted the research team immediately and, depending on logistics, the individual was tagged and released the same or the following day. Upon arrival of the research team, the fishermen would pull the net to the surface, so that the porpoise could be lifted into the fishing boat by hand and placed on foam pads covered with a stretcher made of two poles and tarpaulin.

Three porpoises were caught actively using drifting gillnets in the Wadden Sea. Two boats were used during the capture, with each boat holding two nets (260 m long, 9 m deep, 0.7 mm twine, and 180 mm between the knots). When visual contact with a group of porpoises had been established, nets were deployed from each boat at high speed in front of the porpoises. The boats kept visual contact with the porpoises and circled around the group to make them swim towards the nets. Some individuals were caught in the net immediately, while others would go under the net or under the boat several times until either caught or escaping. As soon as there was any sign of entanglement the boats would rush to the net and keep the porpoise(s) at the surface, disentangle the net, and lift the individual(s) into the boat where they were placed on foam pads as described above.

**Fig. 2:**
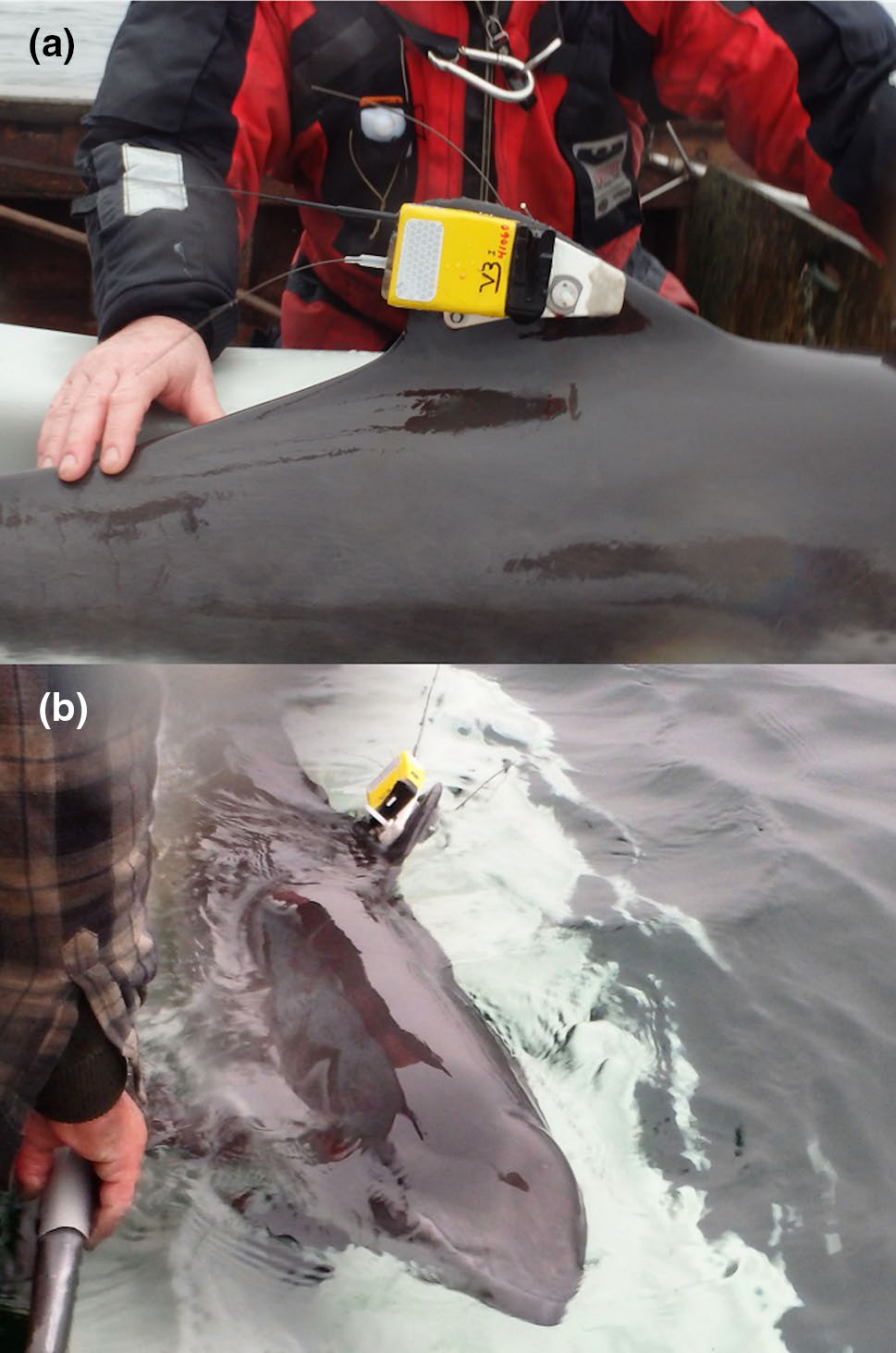
Pictures of the V-tag (version 3) directly after being fitted on the right side of the dorsal fin of a harbour porpoise (**a**) and during release of the animal (**b**). Note the magnesium nut on the front pin, the black GPS unit in the middle of the tag and the small ARGOS, VHF and dive logger hidden in the yellow float material. The ARGOS tag on the left side of the dorsal fin is visible in (**b**), which remained on the porpoise after the V-tag released after about 7–12 days

All caught porpoises were inspected for physical injuries or unusual appearance, while breathing was monitored. A heart rate meter (Polar S810) was placed around the body behind the pectoral fins to monitor whether the heart rate remained between 50 and 200 bpm as recommended by Eskesen et al. (2009). During handling, all porpoises were covered with wet towels and regularly watered down to facilitate breathing and avoid overheating and drying of the skin. When a caught porpoise was deemed large enough (≥ 120 cm standard length) and fit for tagging, two separate tag packages were attached. The first tag was an ARGOS satellite transmitter (SPOT5 weighing 55 g, Wildlife Computers, Redmond, WA, USA) that was fitted with two 5 mm pins trough the left side of the dorsal fin. This tag was intended to remain on the animal for several months to monitor long-term movements of the porpoise (not presented in this study). The second tag (V-tag, Fig. 2) deployed on the right side of the fin consisted of a custom-made high-density closed cell foam package containing a Fastloc GPS (F5G 133A, Sirtrack, Havelock North, New Zealand) and a Time-Depth Recorder (TDR, Lat1800ST, Lotek, Ontario, Canada or a DST F-milli, StarOddi, Reykjavik, Iceland). The GPS unit attempted to acquire and store a location every 3rd min, while the TDR unit registered a depth value every second. Both the GPS and TDR data were used in this study. The V-tag was held in place using a dissolving magnesium bolt on the front pin of the ARGOS tag, while the rear pin from the ARGOS tag was used to stabilise the orientation of the V-tag (Fig. 2a). The dissolving bolt enabled the tag to detach and drift to the surface within approximately 14 days (Table 1). The V-tag also contained a VHF radio transmitter (ATS, Isanti, MN, USA) and a small ARGOS transmitter (SPOT5, Wildlife Computers, Redmond, WA, USA), which were necessary to retrieve the tag after it released from the animal. The V-tag had a weight of 135 g (version 3) or 150 g (version 2), and was slightly positively buoyant in water. Total handling time of each porpoise during the tagging procedure was < 30 min, after which they were released back into the water (Fig. 2b). We did not experience any mortality or unexpected incidences during capture, handling or tagging of the porpoises, and ARGOS data showed that animals continued moving throughout the study area after the V-tag had detached. After the V-tags had released, they were retrieved using satellite positions from the ARGOS tag (accessible in real time from the internet) and VHF signal (short range tracking with R1000 radios, Communications Specialists, http://www.com-spec.com/).

### Horizontal and vertical movement parameters

Horizontal movement data were successfully obtained from all six individuals, but partial tag failure (TDR unit) resulted in missing vertical movement (dive) data for one individual (ID 6: Table 1).

We screened for positional outliers in the GPS data, i.e., when the porpoise moved at an unlikely speed between two consecutive locations (> 15 km h^−1^) and returned to the site, it came from in the subsequent move. With this approach, we removed 331 locations out of 17 175 locations (< 2% of the full GPS data set). Although the GPS units acquired on average one successful location every 15 min, the time between successive locations was highly variable within and among individuals (Table 1, Supplementary Figure S2). Calculation of horizontal movement statistics based on irregular time series can bias estimates (Schick et al. 2008). We, therefore, regularized the GPS tracks by retaining those GPS locations acquired every quarter-hour with a tolerance band of 2 min (every 13–17 min). After positional outliers were removed and the data were regularized, we created individual-specific horizontal movement trajectories using the package “adehabitatLT” in R (Calenge 2006). From each horizontal movement trajectory, we calculated two frequently used movement statistics, namely, movement speed and turning angle. Speed of movement (m s^−1^) was calculated as the Euclidian distance (m) travelled between two consecutive GPS positions divided by the time lag (s) between location attempts. Turning angles (0° to 180°) were calculated as the absolute value of the turning angle between three consecutive GPS locations. Turning angles close to 0° represent directed movements, while values close to 180° represent tortuous movements. Location data collected within 24 h after tagging were discarded to ensure that potential capture/tagging-related effects on movement behaviour were excluded from the data (van Beest et al. 2018). We repeated the above process to create two additional horizontal movement data sets that were regularized using a 30-min interval and a 60-min interval. We did this to evaluate and ensure that the (arbitrarily) chosen 15-min temporal resolution of the data regularization process did not have a major impact on the results.

Data collected by the TDR units were used to create individual-specific time-depth profiles (Supplementary Figure S3). Preliminary screening of the TDR data with the R package “diveMove” (Luque 2007) revealed no drift in the pressure transducers and depth recordings. As such, we did not require a zero-offset correction procedure. As for the location data, all dive data collected within 24 h after tagging were discarded. We defined a dive as any vertical movement exceeding 2 m and lasting at least 10 s (sensu Teilmann et al. 2007). From each time-depth profile, we extracted four vertical movement parameters: dive duration (s), maximum dive depth (m), dive wiggliness, i.e., the absolute vertical distance (m) covered at the bottom of each dive, which is a good movement proxy for prey chasing behaviour (sensu Leos-Barajas et al. 2017), and post-dive duration [the time (s) at the surface between dives]. Besides the full vertical movement data set containing all dives and the four dive parameters, we also created a condensed vertical movement data set, where we only retained dives that took place within a 60-s interval around the timestamp of acquired GPS locations with a 15-min interval. A condensed vertical movement data set was necessary to relate dive behaviour to changing environmental conditions, which is only possible for dive parameters with an associated positional estimate.

### Environmental data

All static and dynamic environmental variables considered here were selected based on results from previous studies on large-scale movement, distribution, and general space use of harbour porpoises (see Table 2 and references therein) and of their main prey species in this part of their distribution (Hedger et al. 2004; Akimova et al. 2016). We considered three static environmental variables including bathymetry, sea bottom slope, and distance to coast (the Euclidian distance to closest land mass including mainland or islands), which were calculated based on a digital elevation model (300 m resolution). We considered five dynamic environmental variables including sea-surface temperature, sea-surface salinity, sea-surface height (the difference in height of sea surface relative to the mean sea height), sea-surface current velocity, and hour of the day. We obtained estimates of sea-surface conditions (top 1 m of water column) through remotely sensed hourly raster data (7 km resolution) freely accessible from the Copernicus Marine Environmental Monitoring Service (CMEMS: http://marine.copernicus.eu/). CMEMS rasters were model prediction values for the Atlantic—European North-West shelf area as derived by the Forecasting Ocean Assimilation Model Atlantic Margin model (von Schuckmann et al. 2016). We appended the value of each environmental variable to the movement data (including both horizontal and vertical movement parameters) using GPS locations and the associated time stamp.

**Table 2:** List of all candidate predictor variables, their unit, and the spatiotemporal resolution of the environmental data used in the current study

Variable name	Unit	Spatial resolution	Temporal resolution	References
Bathymetry	m	300 m	–	Edrén et al. (2010), Gilles et al. (2016), Mikkelsen et al. (2016)
Seabed slope	°	300 m	–	Gilles et al. (2016), Mikkelsen et al. (2016)
Distance to coast	m	300 m	–	Edrén et al. (2010), Gilles et al. (2016), Mikkelsen et al. (2016)
Sea-surface temperature^a^	°C	7 km	Hourly	IJsseldijk et al. (2015), Gilles et al. (2016), Mikkelsen et al. (2016)
Sea-surface salinity^a^	PSU	7 km	Hourly	Edrén et al. (2010), Gilles et al. (2016), Mikkelsen et al. (2016)
Sea-surface height^a^	m	7 km	Hourly	IJsseldijk et al. (2015), Benjamins et al. (2017)
Sea-surface velocity^a^	m s^−1^	7 km	Hourly	IJsseldijk et al. (2015), Mikkelsen et al. (2016)
Hour of the day	h	–	Hourly	IJsseldijk et al. (2015), Gilles et al. (2016), Schaffeld et al. (2016)

References are provided to other studies in which the listed environmental conditions were considered important in explaining movement, distribution or general space use of harbour porpoises in this part of their range (i.e., North-East Atlantic and European shelf waters). Note that this is not intended as an exhaustive literature review

^a^Values derived from the Forecasting Ocean Assimilation Model 7 km Atlantic Margin model (FOAM AMM7) as part of the Copernicus Marine Environment Monitoring Service (CMEMS)

### Statistical analyses

To test for differences in movement patterns among porpoises, we generated individual-specific frequency distributions for each movement parameter followed by analysis of variance (ANOVA) and post hoc paired Tukey HSD tests for repeated measurements.

To quantify the influence of environmental conditions on variation in horizontal and vertical movements, we employed a multi-model inference technique and model averaging (Burnham and Anderson 2002; Burnham et al. 2011) using the R package “MuMIn” (Bartoń 2016). Each movement parameter was fitted as the response variable in a separate linear regression and the environmental variables were fitted as predictor variables. Sea-surface height and hour of the day were fitted as second order polynomials in all models to allow for non-linearity in the response. We calculated the relative variable importance (*w*_+_(*j*)) for each environmental covariate by summing the Akaike’s weights across all possible models where variable *j* occurred. It is generally assumed that the larger the *w*_+_(*j*) the more important variable *j* is for the data being analysed (Burnham and Anderson 2002; Giam and Olden 2016). However, to reduce the risk of drawing ecological inference on potentially uninformative variables (Arnold 2010), we also calculated the 95% confidence interval (CI) of the regression coefficients through model averaging. Environmental variables where the 95% CI of the regression coefficients did not contain 0 were considered to have a biological effect on the movement parameter under investigation.

Preliminary data analyses revealed collinearity within the set of environmental variables considered (Table 2) with bathymetry being strongly correlated with distance to coast (*r *= 0.72), seabed slope (*r *= 0.59), and sea-surface temperature (*r *= − 0.53). Inclusion of correlated predictor variables in model averaging can lead to erroneous results (Cade 2015) and we, therefore, ran different sets of models, where either bathymetry or the combination of distance to coast, seabed slope, and sea-surface temperature was included. We then evaluated which model set performed best given the data by comparing AIC values adjusted for small sample size (AIC_c_). We did not consider tagging site and harbour porpoise ID as covariates in the models or interactions between covariates due to our moderate sample size. As such, our models provide population-averaged effects.

To satisfy statistical assumptions of linear regression, we (1) log_10_ transformed the values of each movement parameter, except for turning angle, to achieve normality; (2) included the corAR1 temporal autocorrelation function to account for dependence among repeated measurements; and (3) incorporated harbour porpoise ID into the VarIdent variance structure to account for differences in residual spread. To do so, models were fitted using generalized least square linear regression (GLS) through the R package “nlme” (Pinheiro et al. 2017). To assess the amount of variation in the data explained by each candidate model, we also calculated a generalized R^2^ suitable for GLS by taking the square of the correlation between the fitted values of the model and the observed values in the data (Zheng and Agresti 2000).

## Results

**Fig. 3:**
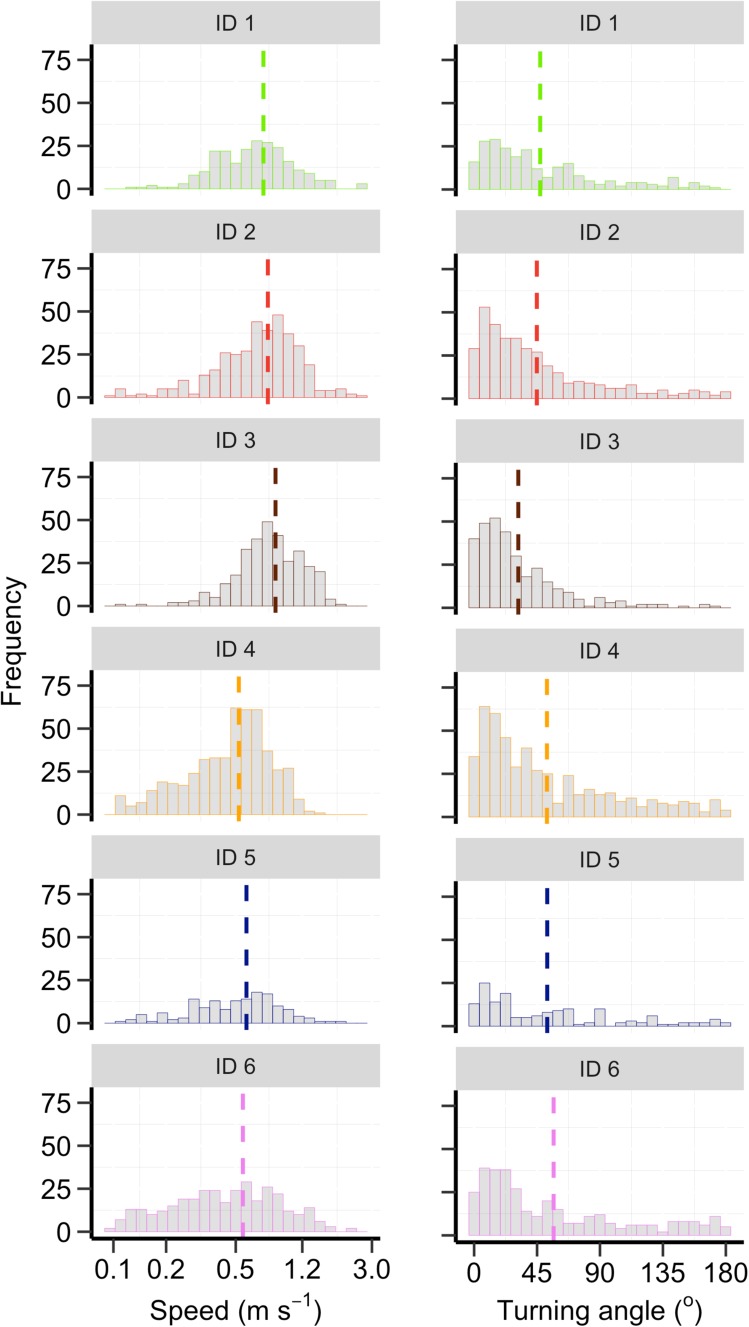
Individual-specific frequency distributions for horizontal movement speed (m s^−1^) and turning angles (°) between successive GPS locations taken at 15-min intervals. Colours of the bars indicate the six different harbour porpoise individuals and the vertical dashed lines indicate individual-specific means of the movement parameter

**Fig. 4:**
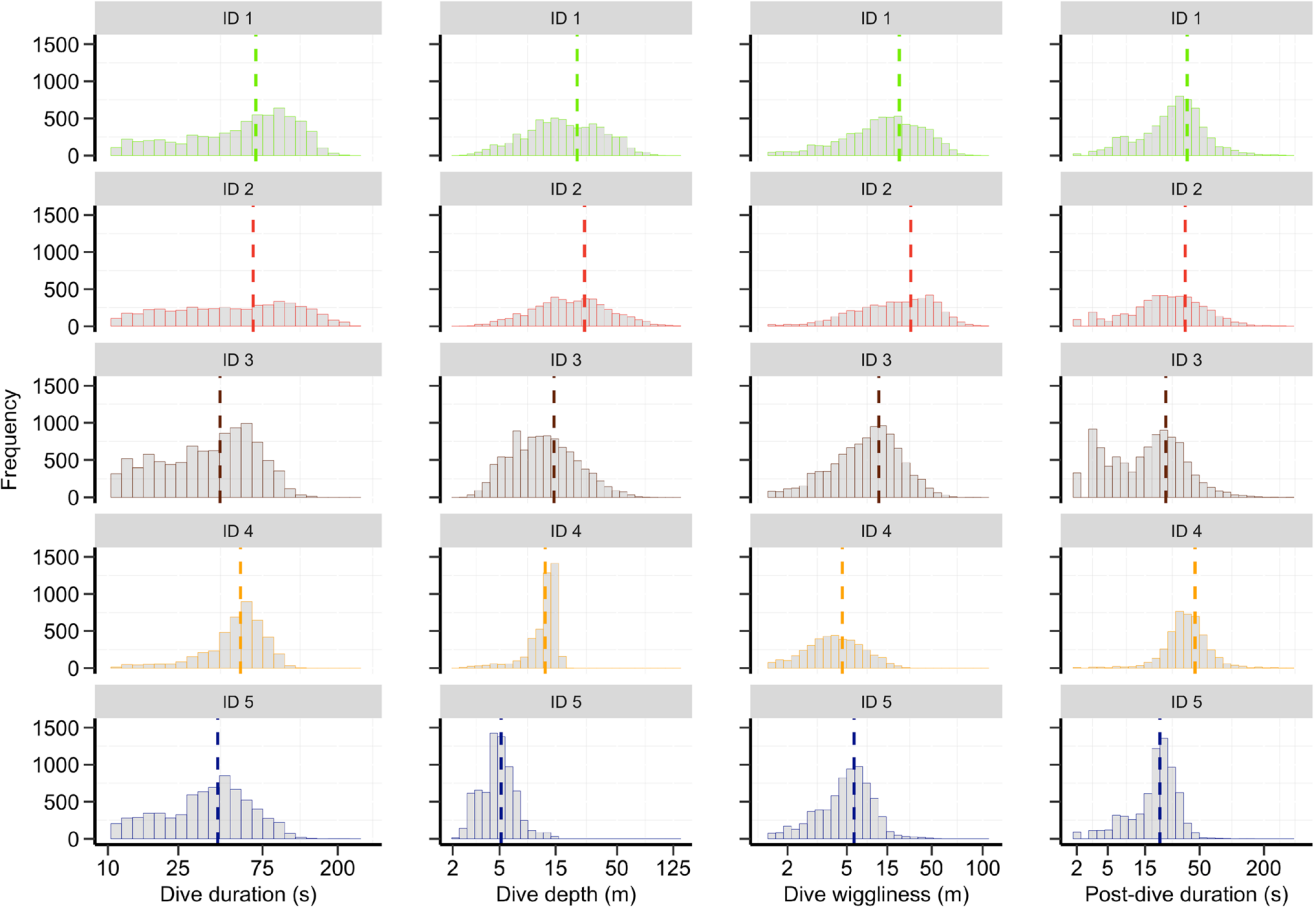
Individual-specific frequency distributions for each vertical movement parameter: dive duration (s), dive depth (m), dive wiggliness (m), and post-dive duration (s). Colours of the bars indicate five different harbour porpoise individuals and the vertical dashed lines indicate individual-specific means of the movement parameter. Note that ID 6 could not be shown here due to missing dive data

The mean horizontal speed moved between successive GPS locations was 0.65 m s^−1^ (min = 0.15 m s^−1^, max = 2.8 m s^−1^) when pooling all individuals together. However, individual differences were observed (Fig. 3; *F*_1,5_ = 26.44, *P* < 0.001). The mean absolute turning angle between successive GPS locations was 43° (min = 0.01°, max = 179.8°), but again, individual differences were found (Fig. 3; *F*_1,5_ = 13.16, *P* < 0.001). Although the mean movement speed and turning angle changed slightly as the interval between GPS locations increased from 15 to 30 and 60-min, individual differences remained (Supplementary Figure S4). The mean dive duration was 53 s (min = 10.1 s, max = 250.0 s) when pooling all individuals together, yet individual differences were observed (Fig. 4; Tukey HSD: *P* < 0.001 for all comparisons). Individual differences (Tukey HSD: *P* < 0.001) were also found for dive depth (mean = 15.5 m, min = 2.25 m, max = 151.5 m; Fig. 4) and dive wiggliness (mean = 14 m, min = 0.9 m, max = 90.6 m, Fig. 4). The mean post-dive duration was 39 s (min = 2 s, max = 309 s; Fig. 4), which also varied among individuals (Tukey HSD: *P* < 0.001).

**Fig. 5:**
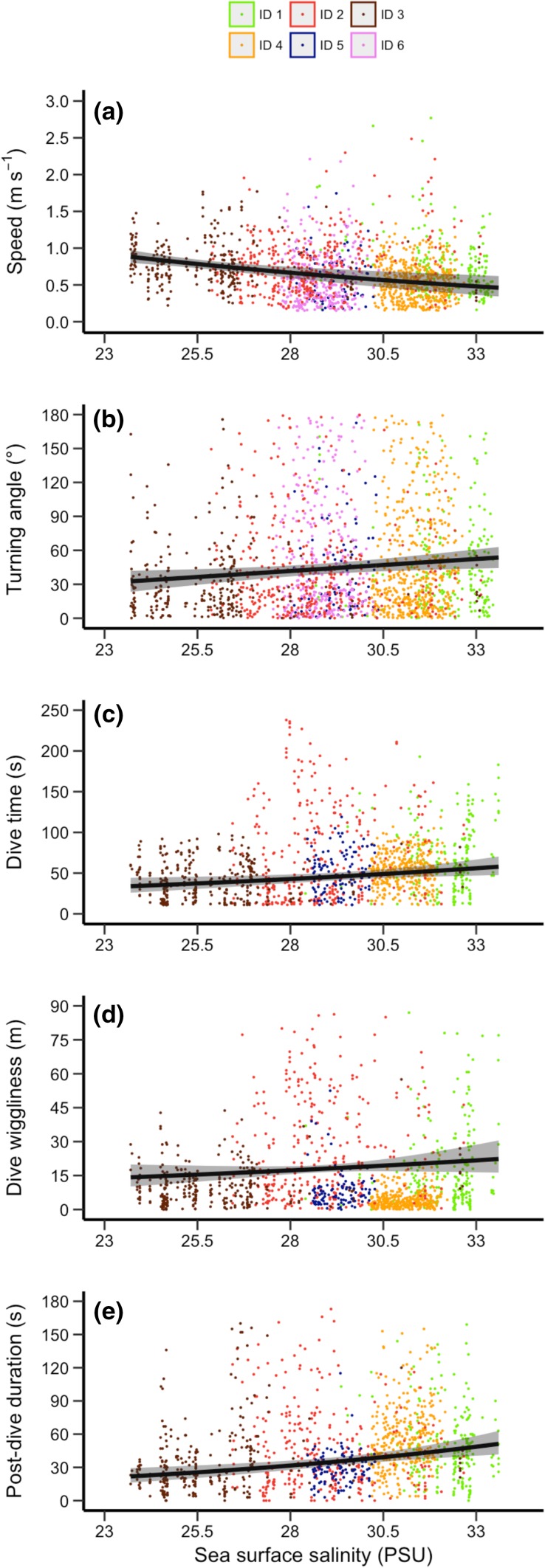
Predicted effect of sea-surface salinity on harbour porpoise movement speed (**a**), turning angle (**b**), dive duration (**c**), dive wiggliness (**d**), and post-dive duration (**e**). Predictions were made while keeping other variables in the full model constant at their mean value. Black lines show the back-transformed (from log_10_ scale) predicted marginal (population-level) effect with shaded areas indicating the 95% CI around the mean, and coloured points show raw data values for each porpoise. Note that ID 6 could not be shown on panels **c**–**e** due to missing dive data

Sea-surface salinity was the most informative dynamic environmental condition that influenced porpoise movements (Table 3). Indeed, sea-surface salinity had consistently high variable importance (*w*_+_(*j*)) with 95% CI of regression coefficients excluding 0 in most models. Speed of horizontal movements decreased with increasing sea-surface salinity (Fig. 5a), while absolute turning angles increased (Fig. 5b). These patterns remained irrespective of the temporal resolution of the GPS data used to calculate horizontal movement parameters (Supplementary Table S1). The importance of sea-surface salinity was also present in the vertical movement models as dives became substantially longer (Fig. 5c), wigglier (Fig. 5d) and with longer post-dive resting intervals (Fig. 5e) as porpoises moved into more saline waters. Dive depth did not vary as a function of sea-surface salinity (Table 3).

**Table 3:** Model averaging results for each of the six movement models that included bathymetry, but not distance to coast, slope, and sea-surface temperature due to collinearity

Movement model	Parameter name	Beta	95% CI	*w*_+_(j)	Mean *R*^2^
			Lower, upper		(min, max)
Speed (m s^−1^)	(Intercept)	− 0.223	− 0.239, − 0.207	NA	0.29
	Bathymetry	0.051	0.034, 0.068	**1**	(0.12, 0.32)
	Sea-surface salinity	− 0.070	− 0.084, − 0.055	**1**	
	Sea-surface height	0.010	− 0.255, 0.274	0.16	
	Sea-surface height^2^	− 0.041	− 0.353, 0.270		
	Sea-surface velocity	0.010	− 0.008, 0.028	0.68	
	Hour of the day	− 0.589	− 1.309, 0.131	0.84	
	Hour of the day^2^	− 0.332	− 0.959, 0.295		
Turning angle (°)	(Intercept)	44.61	42.5, 46.9	NA	0.08
	Bathymetry	− 1.87	− 4.82, 1.09	0.75	(0.02, 0.09)
	Sea-surface salinity	5.42	3.31, 7.52	**1**	
	Sea-surface height	− 3.31	− 27.1, 20.4	0.14	
	Sea-surface height^2^	− 2.41	− 23.1, 18.3		
	Sea-surface velocity	− 0.294	− 1.986, 1.398	0.32	
	Hour of the day	− 52.76	− 129.7, 24.2	0.86	
	Hour of the day^2^	− 53.78	− 136.1, 28.5		
Dive duration (s)	(Intercept)	1.672	1.648, 1.696	NA	0.31
	Bathymetry	0.045	0.015, 0.075	**0.97**	(0.14, 0.36)
	Sea-surface salinity	0.055	0.030, 0.078	**1**	
	Sea-surface height	− 0.483	− 1.187, 0.221	0.53	
	Sea-surface height^2^	0.456	− 0.210, 1.122		
	Sea-surface velocity	0.014	− 0.010, 0.038	0.42	
	Hour of the day	0.447	− 0.249, 1.144	0.26	
	Hour of the day^2^	0.271	− 0.486, 1.027		
Dive depth (m)	(Intercept)	1.096	1.069, 1.121	NA	
	Bathymetry	0.156	0.122, 0.189	**1**	
	Sea-surface salinity	0.014	− 0.010, 0.038	0.6	
	Sea-surface height	0.063	− 0.304, 0.4313	0.29	
	Sea-surface height^2^	0.121	− 0.346, 0.588		
	Sea-surface velocity	− 0.011	− 0.034, 0.012	0.6	
	Hour of the day	0.010	− 0.235, 0.256	0.17	
	Hour of the day^2^	-0.050	− 0.390, 0.289		
Dive wiggliness (m)	(Intercept)	0.882	0.845, 0.918	NA	0.25
	Bathymetry	0.226	0.184, 0.266	**1**	(0.11, 0.29)
	Sea-surface salinity	0.056	0.011, 0.110	**0.96**	
	Sea-surface height	0.298	− 1.002, 1.599	0.78	
	Sea-surface height^2^	− 1.688	− 2.991, 0.385		
	Sea-surface velocity	0.045	− 0.006, 0.095	0.78	
	Hour of the day	1.250	0.018, 2.481	0.56	
	Hour of the day^2^	0.533	− 0.728, 1.795		
Post-dive duration (s)	(Intercept)	1.546	1.513, 1.577	NA	0.23
	Bathymetry	− 0.001	− 0.023, 0.021	0.27	(0.11, 0.27)
	Sea-surface salinity	0.092	0.056, 0.128	**1**	
	Sea-surface height	− 0.217	− 1.037, 0.602	0.58	
	Sea-surface height^2^	0.528	− 0.624, 1.680		
	Sea-surface velocity	− 0.009	− 0.037, 0.020	0.43	
	Hour of the day	0.149	− 0.567, 0.864	0.23	
	Hour of the day^2^	0.019	− 0.481, 0.519		

Regression coefficients (Beta) and 95% CI are model-averaged estimates of each environmental variable calculated over the candidate model set. The relative variable importance (w_+_(*j*)) is the sum of the Akaike’s weights across all possible models where variable *j* occurred. The w_+_(*j*) is provided for all covariates and those in bold indicate that the 95% CI does not overlap with 0. The mean (min, max) generalized *R*^2^ value is provided and calculated from the full set of candidate models. Note that the environmental variables “Sea-surface height” and “Hour of the day” were fitted as second order polynomials, and therefore, two regression coefficients (Beta) and 95% CIs are provided but only one w_+_(*j*)

**Fig. 6:**
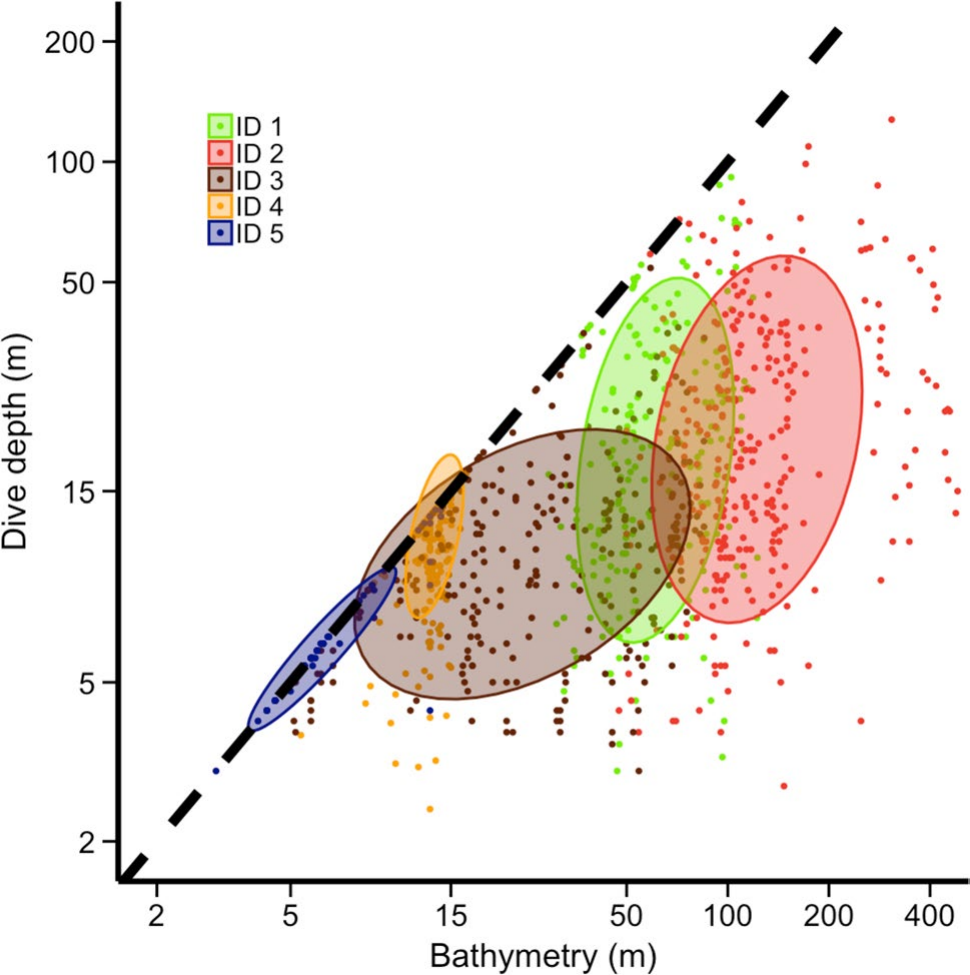
Plot of bathymetry versus individual-specific dive depth. Coloured dots close to the dashed black line indicate dives down to the seabed and coloured eclipses surround 65% of the dives taken around the mean dive depth of each porpoise. Note that ID 6 could not be shown due to missing dive data

Models that included bathymetry consistently performed better than models with distance to coast, slope, and sea-surface temperature as determined by AIC_c_ (see Supplementary Tables S2 and S3 for model averaging results with distance to coast, slope, and sea-surface temperature). Horizontal movement speed increased with increasing bathymetry but we found no effect on turning angles (Table 3). Again, these patterns remained irrespective of the temporal resolution of the GPS data used to calculate horizontal movement parameters (Supplementary Table S1). As porpoises moved into deeper water dives became longer, wigglier, and deeper (Fig. 6) but without a strong change in post-dive duration (Table 3).

All movement models fulfilled the main assumptions of linear regression analyses as revealed by inspection of model residuals (Supplementary Figures S5–S10). The generalized *R*^2^ of the candidate set of models ranged between 0.02 and 0.36 with the least amount of variation explained in turning angles and most of the variation explained in dive duration (Table 3).

## Discussion

Our study is the first to quantify fine-scale movements of individual harbour porpoises equipped with both GPS and dive tags, and to assess how variation in movement relates to environmental conditions in the Danish part of the North Sea. We found that bathymetry and sea-surface salinity in particular were the most important environmental drivers of porpoise fine-scale movements. It is commonly accepted that marine predators follow the abundance and distribution of their prey, which is especially applicable to porpoises as they need to forage regularly to meet their high metabolic demands (Wisniewska et al. 2016). Porpoises reduced their speed, turned more, and made longer, wigglier dives with longer post-dive resting intervals as they moved into more saline waters (Fig. 5). Such movement parameters are indicative of foraging behaviour (Leos-Barajas et al. 2017) and corroborates our initial expectation that sea-surface salinity is a good environmental indicator of potentially important feeding areas for porpoises, at least in the Danish part of the North Sea. The importance of bathymetry on porpoise movements, and on dive behaviour especially, may point to differences in hunting strategy between tracked porpoise individuals. We found that some individuals consistently dove down to the seabed which is indicative of a hunting strategy focussed on demersal fish species, while other individuals covered a much wider bathymetry gradient representing a more generalist-opportunistic hunting strategy targeting both pelagic and demersal fish. However, this result could be confounded by differences in prey assemblages and physical conditions between the two tagging sites. Robust inference on how the movement behaviour and hunting strategy of porpoises change along the bathymetry gradient would require tracking of individuals in areas with known prey availability and assemblages, which is a major and general challenge when studying free-ranging and highly mobile marine mammals.

Besides the consistent importance of bathymetry and sea-surface salinity on various fine-scale movement parameters, we found little support for biological effects of sea-surface height, current velocity, and time of day, even though these environmental conditions have previously been shown to influence porpoise habitat use and occurrence (Table 2). Tracking fine-scale movements of a larger number of individuals, and over a broader geographical area, is needed to evaluate whether our results are generally applicable or specific to these data or regions. An increase in sample size would also allow for the inclusion of sex, age, and body size as predictor variables. We were unable to consider these intrinsic variables, as well as other potential drivers of fine-scale movement such as, e.g., predation risk and human disturbance into our analyses, due to limited sample size and lack of data on the presence of predators and disturbances. Doing so would likely explain additional variation in the observed movement patterns and is, therefore, an important focus area for future tracking studies.

The European Atlantic shelf waters are used extensively by porpoises but also by humans for fishing, oil and gas extraction, shipping, and offshore wind farm development. Independent case studies have highlighted two common impacts of such anthropogenic activities on harbour porpoises, namely, behavioural alterations (Tougaard et al. 2012; Dyndo et al. 2015) and direct mortality or injury of individuals (Vinther and Larsen 2004; Lucke et al. 2009). To assist in the conservation of harbour porpoises, there has been increased effort to develop predictive simulation models to assess any consequences of anthropogenic stressors and disturbances on individuals and populations (Nabe-Nielsen et al. 2014; King et al. 2015; Aarts et al. 2016; van Beest et al. 2017). Such simulation models are valuable tools to highlight areas of potential conflict with current or planned anthropogenic activities and to evaluate the effectiveness of potential mitigation measures. However, data on fine-scale movement behaviour of harbour porpoises in these models are currently limited (Nabe-Nielsen et al. 2014; van Beest et al. 2017), assumed (Aarts et al. 2016) or absent (King et al. 2015). The data and results of our study thus serve as a valuable baseline to further refine these, and future, movement-based simulation models.

## Electronic supplementary material

Below is the link to the electronic supplementary material.

Supplementary material 1 (PDF 2621 kb)
