# Supplementary material for: Environmental drivers of harbour porpoise fine-scale movements
Source: Mar Biol. 2018 Apr 30;165(5):95. doi: 10.1007/s00227-018-3346-7 (PMC5924767; doi:10.1007/s00227-018-3346-7)
Supplement: Supplementary file 1 — Supplementary material 1 (PDF 2621 kb) [file 227_2018_3346_MOESM1_ESM.pdf]

The following supplement accompanies the manuscript:

Environmental drivers of harbour porpoise fine-scale movements

Floris M. van Beest[1,*], Jonas Teilmann[1], Rune Dietz[1], Anders Galatius[1], Lonnie Mikkelsen[1], Dominique Stalder[1], Signe Sveegaard[1], Jacob Nabe-Nielsen[1]

[1] Department of Bioscience, Aarhus University, Frederiksborgvej 399, DK-4000 Roskilde, Denmark
[*] Corresponding author: flbe@bios.au.dk, ORCID: 0000-0002-5701-4927

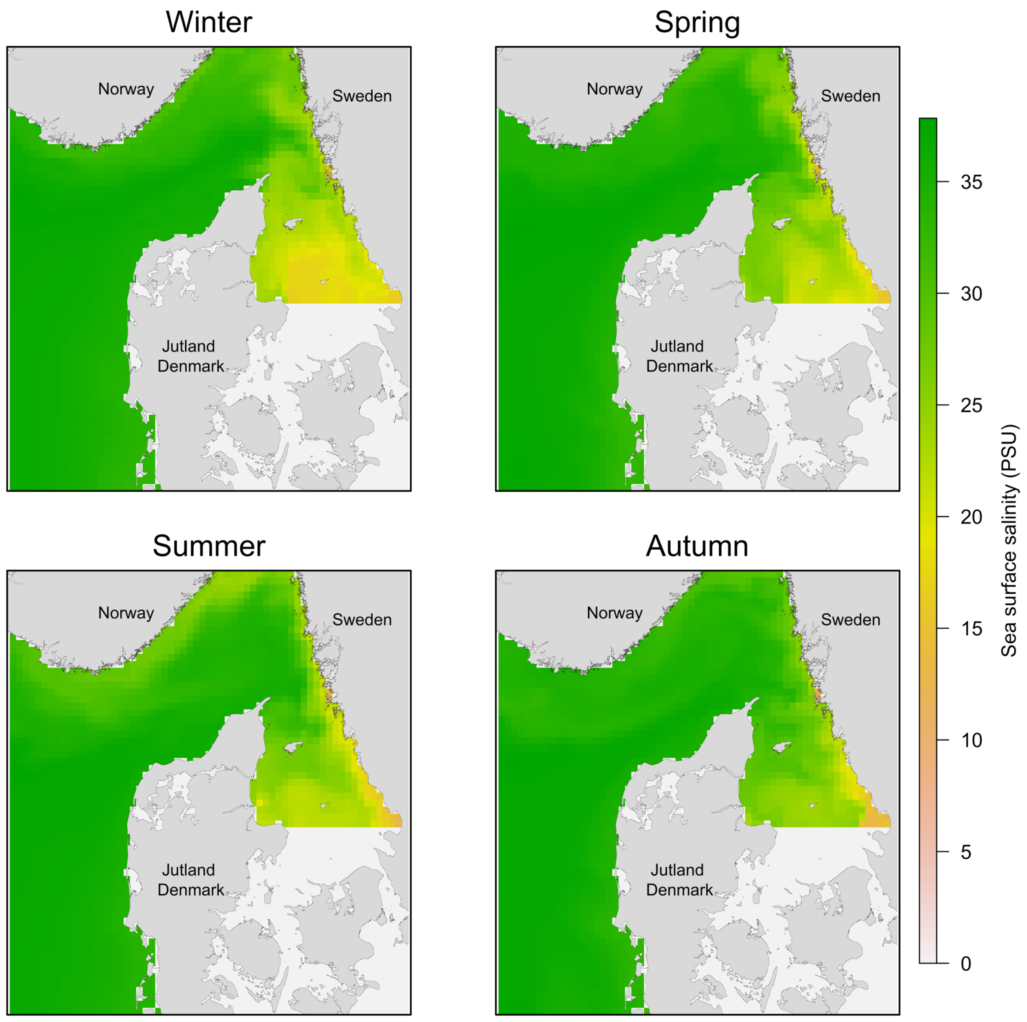

Fig. S1: Mean sea surface salinity (Practical Salinity Unit; PSU) in the study area across seasons in 2015. Winter is defined as December–February, spring as March–May, summer as June–August, and autumn as September–November. Salinity values south of Kattegat are not plotted, as these are not included in the Forecasting Ocean Assimilation Model 7km Atlantic Margin model (FOAM AMM7) as part of the Copernicus Marine Environment Monitoring Service (CMEMS), which we used for the data analyses.

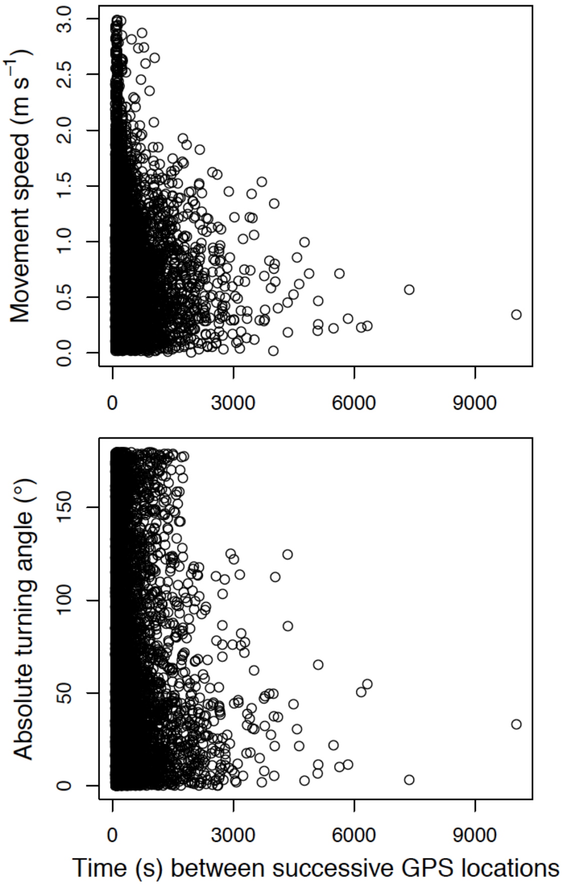

Fig. S2: Harbour porpoise movement speed and absolute turning angle as a function of time between successive GPS locations calculated from six irregular GPS movement trajectories from Danish waters.

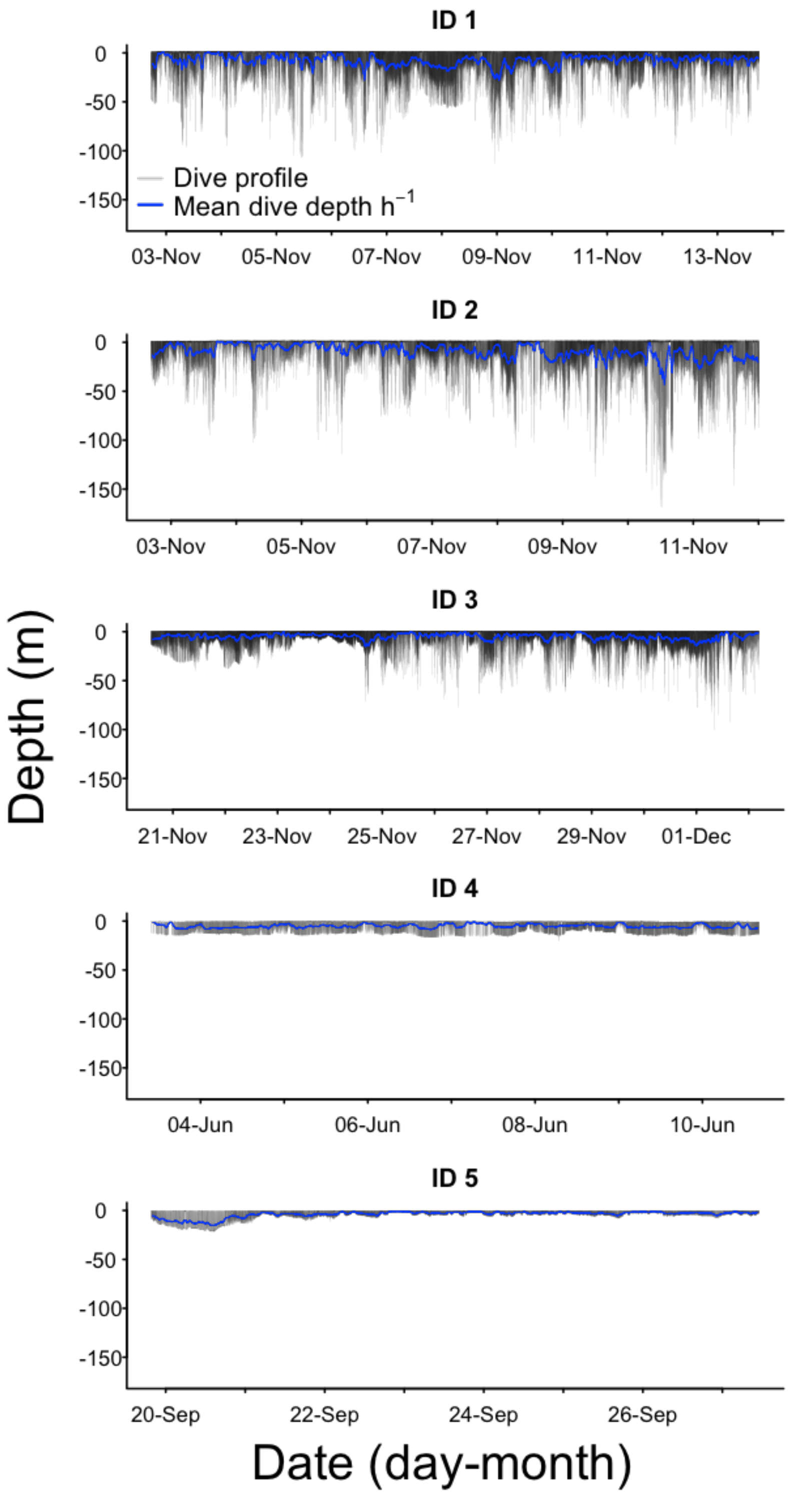

Fig. S3: Time-dive profiles (including data collected 24-hours after tagging that were not included in the analyses) for five (out of six) harbour porpoises tagged for this study. Individual dives are shown in dark grey with the rolling mean dive depth h^{-1} shown in blue. Note that ID 6 did not have a functioning TDR-unit and as such the dive profile could not be plotted.

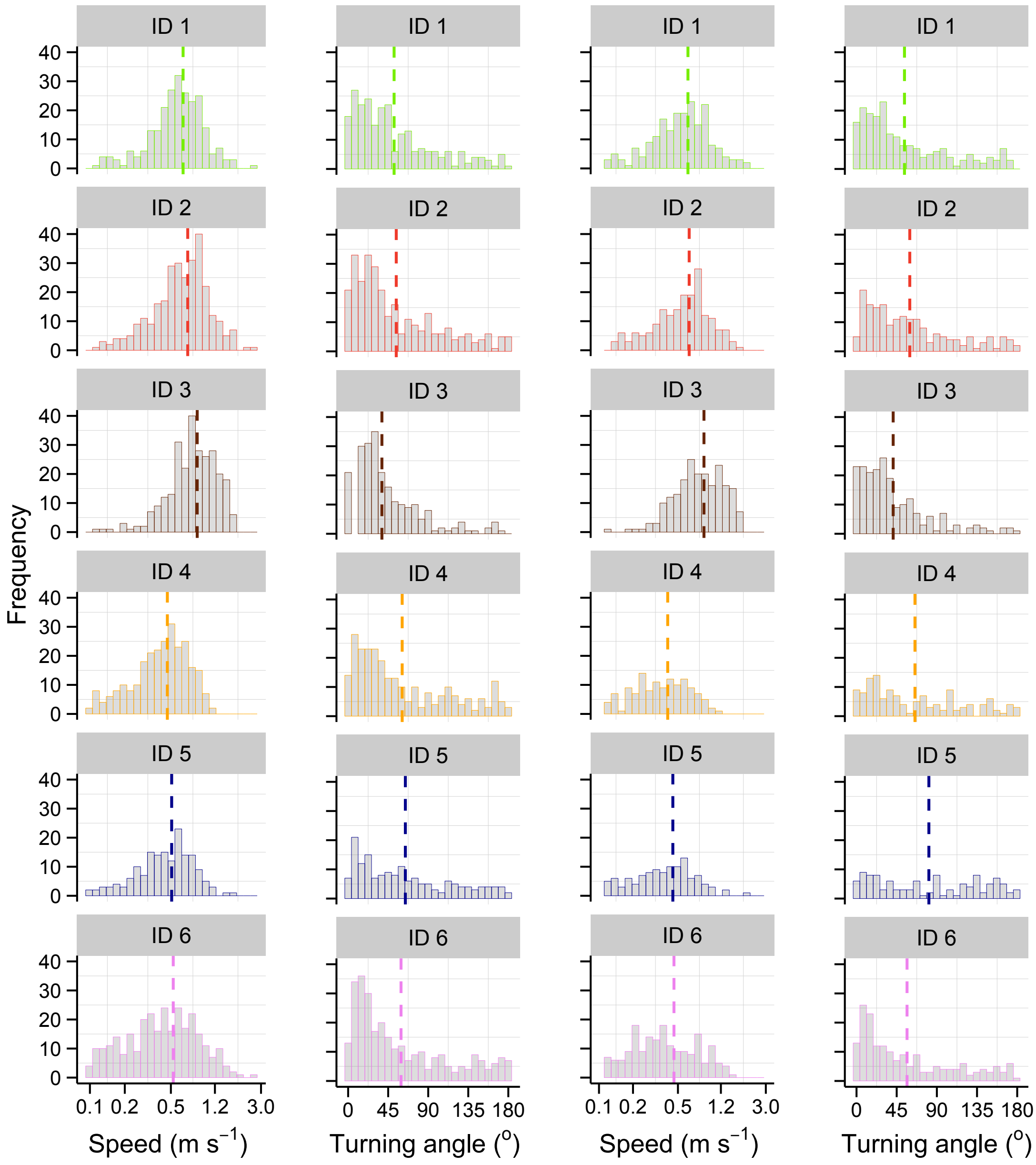

Fig. S4: Frequency distribution of horizontal movement speed (m s^{-1}) and turning angles (°) between successive GPS locations taken at 30-minute (first 2 columns) and 60-minute (last 2 columns) intervals. Colours of the bars and vertical dashed lines indicate the six different harbour porpoises and the individual-specific mean of the movement parameter. The mean speed moved between successive GPS locations was 0.58 m s^{-1} (min = 0.11, max = 2.6) and 0.51 m s^{-1} (min = 0.11, max = 2.6) for the 30- and 60 minute interval respectively with significant differences between individuals ($F_{1,5}$ = 38.55, P < 0.001 and $F_{1,5}$ = 33.15, P < 0.001 respectively). The mean absolute turning angle between successive GPS locations was 57° (min = 0.02, max = 179.9) and 60° (min = 0.01, max = 179.4) for the 30- and 60 minute interval respectively with significant differences between individuals ($F_{1,5}$ = 13.27, P < 0.001 and $F_{1,5}$ = 10.38, P < 0.001 respectively).

Table S1. Results of the model averaging procedure for horizontal movement speed and turning angles using the GPS data set with 30-minute interval (A and B) and a 60-minute interval (C and D). Regression coefficients (Beta) and 95% CI are model-averaged estimates of each environmental variable were calculated over the candidate model set. The relative variable importance ($w_+(j)$) is provided for all covariates and those in bold highlight a potential biological effect (i.e. 95% CI does not overlap with 0) on the associated movement parameter.

Table	Movement model	Parameter name	Beta	95% CI lower, upper	w_+(j)	mean R^2 (min, max)
A	Speed ($m\ s^{-1}$)	(Intercept)	-0.244	-0.261, -0.227	NA	0.23
		Bathymetry	0.049	0.031, 0.066	**1**	(0.10, 0.25)
		Sea surface salinity	-0.071	-0.086, -0.054	**1**	
		Sea surface height	0.169	-0.373, 0.711	0.5	
		Sea surface height^2	-0.273	-0.952, 0.406		
		Sea surface velocity	0.001	-0.008, 0.009	0.27	
		Hour of the day	-0.123	-0.576, 0.331	0.36	
		Hour of the day^2	-0.128	-0.611, 0.356		
B	Turning angle (°)	(Intercept)	50.458	47.698, 53.217	NA	0.06
		Bathymetry	-0.353	-2.282, 1.576	0.33	(0.02, 0.08
		Sea surface salinity	4.959	2.344, 7.573	**1**	
		Sea surface height	-34.797	-136.775, 67.180	0.47	
		Sea surface height^2	39.517	-69.668, 148.701		
		Sea surface velocity	-0.127	-1.771, 1.517	0.28	
		Hour of the day	-70.325	-168.136, 27.485	**1**	
		Hour of the day^2	-184.699	-287.178, -82.219		
C	Speed ($m\ s^{-1}$)	(Intercept)	-0.258	-0.283, -0.232	NA	0.24
		Bathymetry	0.040	0.014, 0.066	**0.98**	(0.13, 0.26)
		Sea surface salinity	-0.059	-0.081, -0.036	**1**	
		Sea surface height	0.104	-0.369, 0.578	0.27	
		Sea surface height^2	-0.067	-0.441, 0.307		
		Sea surface velocity	-0.004	-0.020, 0.012	0.36	
		Hour of the day	-0.071	-0.437, 0.295	0.21	
		Hour of the day^2	-0.010	-0.310, 0.289		
D	Turning angle (°)	(Intercept)	53.380	49.791, 56.968	NA	0.05
		Bathymetry	2.037	-1.583, 5.657	0.4	(0.03, 0.07)
		Sea surface salinity	5.230	1.831, 8.628	**0.96**	
		Sea surface height	-77.595	-186.463, 31.273	0.28	
		Sea surface height^2	35.208	-76.023, 146.439		
		Sea surface velocity	-1.878	-5.664, 1.908	0.37	
		Hour of the day	-44.625	-145.441, 56.191	0.82	
		Hour of the day^2	-136.256	-240.847, -31.664		

Table S2. Result of the model averaging procedure for each of the six movement models that included distance to coast, slope and sea surface temperature (but not bathymetry due to collinearity). Regression coefficients (Beta) and 95% CI are model-averaged estimates of each environmental variable that were calculated over the candidate model set. The relative variable importance ($w_+(j)$) is provided for all covariates and those in bold indicate a potential biological effect (i.e. when 95% CI does not overlap with 0). The mean (min, max) generalised R^2 value is provided and calculated from the full set of candidate models. Note that the environmental variables "Sea surface height" and "Hour of the day" were fitted as second order polynomials and therefore two regression coefficients (beta) and 95% CIs are provided but only one $w_+(j)$.

**Movement model**	**Parameter name**	**Beta**	**95% CI lower, upper**	w_+**(j)**	**mean R^2 (min, max)**
Speed (m s^{-1})	(Intercept)	-0.233	-0.249, -0.217	NA	0.30 (0.11, 0.36)
	Slope	0.002	-0.011, 0.016	0.28	
	Distance to coast	0.022	0.005, 0.040	**0.88**	
	Sea surface temperature	-0.049	-0.071, -0.026	**1**	
	Sea surface salinity	-0.044	-0.063, -0.025	**1**	
	Sea surface height	-0.111	-0.775, 0.552	0.13	
	Sea surface height^2	-0.101	-0.730, 0.528		
	Sea surface velocity	0.018	0.003, 0.033	**0.85**	
	Hour of the day	-0.891	-1.453, -0.330	0.28	
	Hour of the day^2	-0.377	-0.982, 0.227		
Turning angle (°)	(Intercept)	45.721	43.325, 48.117	NA	0.12 (0.07, 0.14)
	Slope	1.878	-0.403, 4.160	0.57	
	Distance to coast	-2.862	-5.853, -0.068	**0.71**	
	Sea surface temperature	5.926	1.907, 9.944	**0.96**	
	Sea surface salinity	2.762	0.024, 5.500	**0.71**	
	Sea surface height	33.774	-74.917, 142.464	0.18	
	Sea surface height^2	-45.574	-149.003, 57.855		
	Sea surface velocity	0.076	-2.494, 2.647	0.28	
	Hour of the day	-48.121	-140.982, 44.741	**0.88**	
	Hour of the day^2	-129.423	-224.611, -34.235		
Dive duration (s)	(Intercept)	1.667	1.644, 1.690	NA	0.32 (0.15, 0.36)
	Slope	0.009	-0.015, 0.034	0.33	
	Distance to coast	0.050	0.021, 0.079	**1**	
	Sea surface temperature	0.031	-0.012, 0.075	0.67	
	Sea surface salinity	0.052	0.017, 0.085	**0.97**	
	Sea surface height	-0.305	-1.012, 0.403	0.4	
	Sea surface height^2	0.490	-0.173, 1.152		
	Sea surface velocity	0.016	-0.007, 0.040	0.47	
	Hour of the day	0.492	-0.202, 1.187	0.34	
	Hour of the day^2	0.359	-0.378, 1.096		

Dive depth (m)	(Intercept)	1.095	1.070, 1.118	NA	0.51
	Slope	0.015	-0.009, 0.038	0.42	(0.28, 0.53)
	Distance to coast	0.118	0.093, 0.143	**1**	
	Sea surface temperature	-0.054	-0.177, 0130	0.44	
	Sea surface salinity	0.079	0.054, 0.103	**1**	
	Sea surface height	0.329	-0.180, 0.838	**0.72**	
	Sea surface height^2	0.604	0.117, 1.090		
	Sea surface velocity	-0.008	-0.025, 0.009	0.35	
	Hour of the day	-0.004	-0.527, 0.519	0.15	
	Hour of the day^2	-0.199	-0.748, 0.350		
Dive wiggliness (m)	(Intercept)	0.883	0.846, 0.918	NA	0.26
	Slope	0.078	-0.021, 0.175	0.62	(0.11, 0.29)
	Distance to coast	0.158	0.112, 0.203	**1**	
	Sea surface temperature	-0.062	-0.125, 0.002	0.75	
	Sea surface salinity	-0.049	-0.110, 0.011	0.61	
	Sea surface height	0.039	-1.301, 1.379	0.31	
	Sea surface height^2	-1.003	-2.258, 0.251		
	Sea surface velocity	0.064	0.025, 0.102	**0.98**	
	Hour of the day	0.508	-0.714, 1.731	0.23	
	Hour of the day^2	0.660	-0.585, 1.905		
Post-dive duration (s)	(Intercept)	1.554	1.520, 1.587	NA	0.22
	Slope	0.008	-0.028, 0.043	0.29	(0.11, 0.23)
	Distance to coast	0.034	-0.001, 0.069	0.67	
	Sea surface temperature	-0.016	-0.058, 0.026	0.34	
	Sea surface salinity	0.092	0.052, 0.131	**1**	
	Sea surface height	-0.525	-1.517, 0.467	0.68	
	Sea surface height^2	0.947	-0.021, 1.915		
	Sea surface velocity	-0.019	-0.052, 0.013	0.42	
	Hour of the day	0.605	-0.396, 1.606	0.21	
	Hour of the day^2	0.060	-0.978, 1.098		

Table S3. Results of the model averaging procedure with model sets that included distance to coast, slope and sea surface temperature (but not bathymetry due to collinearity) for horizontal movement speed and turning angles using the GPS data set with 30-minute interval (A and B) and a 60-minute interval (C and D). Regression coefficients (Beta) and 95% CI are model-averaged estimates of each environmental variable were calculated over the candidate model set. The relative variable importance ($w_+(j)$) is provided for all covariates and those in bold highlight a potential biological effect (i.e. 95% CI does not overlap with 0) on the associated movement parameter. The mean (min, max) value of the generalised R^2 is provided and calculated from the full set of candidate models. Note that the environmental variables "Sea surface height" and "Hour of the day" were fitted as second order polynomials and therefore two regression coefficients (beta) and 95% CIs are provided but only one $w_+(j)$.

**Table**	**Movement model**	**Parameter name**	**Beta**	**95% CI** lower, upper	w_+**(j)**	**mean R^2** (min, max)
A	Speed (m s^{-1})	(Intercept)	-0.254	-0.271, -0.237	NA	0.24 (0.11, 0.27)
		Slope	0.004	-0.009, 0.018	0.31	
		Distance to coast	0.029	0.009, 0.048	**0.96**	
		Sea surface temperature	-0.046	-0.069, -0.024	**1**	
		Sea surface salinity	-0.052	-0.070, -0.033	**1**	
		Sea surface height	0.121	-0.489, 0.732	0.16	
		Sea surface height^2	-0.262	-0.859, 0.335		
		Sea surface velocity	0.003	-0.013, 0.020	0.28	
		Hour of the day	-0.433	-0.956, 0.090	0.45	
		Hour of the day^2	-0.319	-0.882, 0.244		
B	Turning angle (°)	(Intercept)	51.358	48.53, 54.11	NA	0.07 (0.04, 0.08)
		Slope	-0.525	-2.936, 1.885	0.29	
		Distance to coast	-1.814	-5.507, 1.879	0.4	
		Sea surface temperature	4.412	0.837, 7.985	**0.86**	
		Sea surface salinity	4.245	0.996, 7.493	**0.93**	
		Sea surface height	-38.442	-148.98, 72.10	0.17	
		Sea surface height^2	43.971	-66.29, 154.23		
		Sea surface velocity	0.043	-3.153, 3.239	0.28	
		Hour of the day	-64.580	-161.57, 32.41	**0.99**	
		Hour of the day^2	-181.606	-280.62, -82.59		
C	Speed (m s^{-1})	(Intercept)	-0.274	-0.297, -0.252	NA	0.26 (0.12, 0.28)
		Slope	-0.008	-0.024, 0.009	0.36	
		Distance to coast	0.029	0.003, 0.055	**0.81**	
		Sea surface temperature	-0.066	-0.096, -0.036	**1**	
		Sea surface salinity	-0.043	-0.068, -0.018	**0.99**	
		Sea surface height	0.077	-0.538, 0.692	0.12	
		Sea surface height^2	0.093	-0.481, 0.666		
		Sea surface velocity	-0.010	-0.030, 0.011	0.35	
		Hour of the day	-0.418	-0.936, 0.101	0.3	
		Hour of the day^2	-0.036	-0.632, 0.560		

D	Turning angle (°)	(Intercept)	54.747	51.05, 58.44	NA	0.08 (0.04, 0.1)
		Slope	1.367	-1.814, 4.548	0.35	
		Distance to coast	2.782	-1.592, 7.156	0.48	
		Sea surface temperature	6.551	1.770, 11.331	**0.94**	
		Sea surface salinity	3.457	-0.466, 7.380	0.63	
		Sea surface height	-34.266	-148.68, 80.15	0.14	
		Sea surface height^2	-2.556	-117.58, 112.46		
		Sea surface velocity	-0.651	-4.504, 3.20	0.29	
		Hour of the day	-47.123	-147.06, 52.81	**0.79**	
		Hour of the day^2	-130.576	-233.88, -27.27		

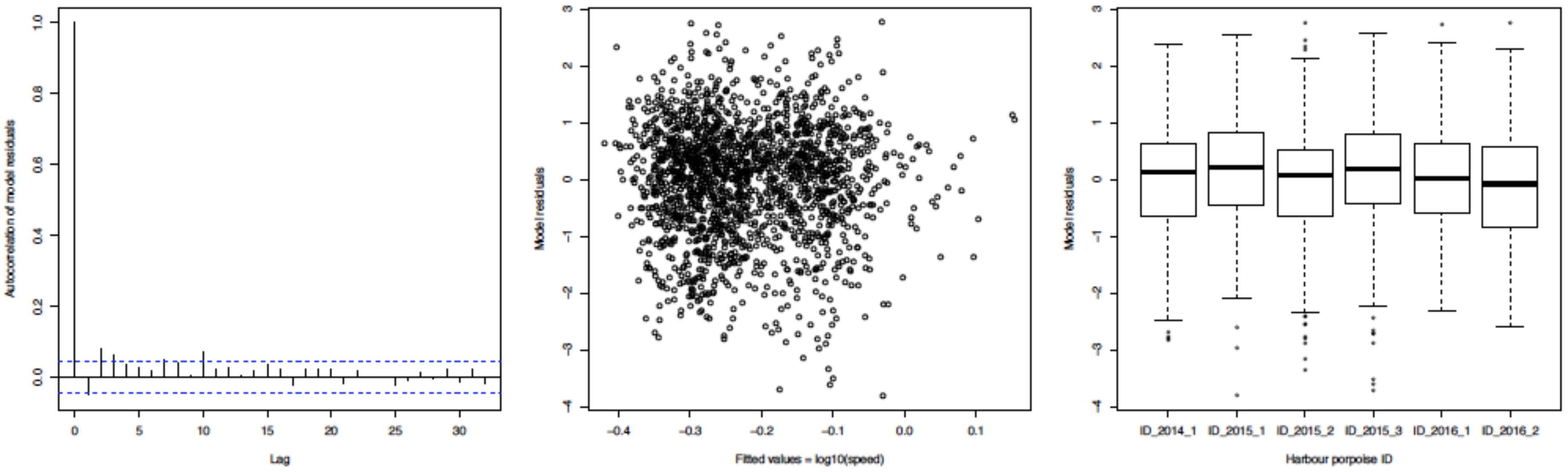

Fig. S5: Model validation plots to assess if the assumptions of linear regression for the horizontal movement speed analysis are fulfilled. Left panel shows the autocorrelation of model residuals (independence of observations), the middle panel shows the fitted values versus model residuals (normality) and the right panel shows the spread of model residuals across porpoise individuals (homogeneity).

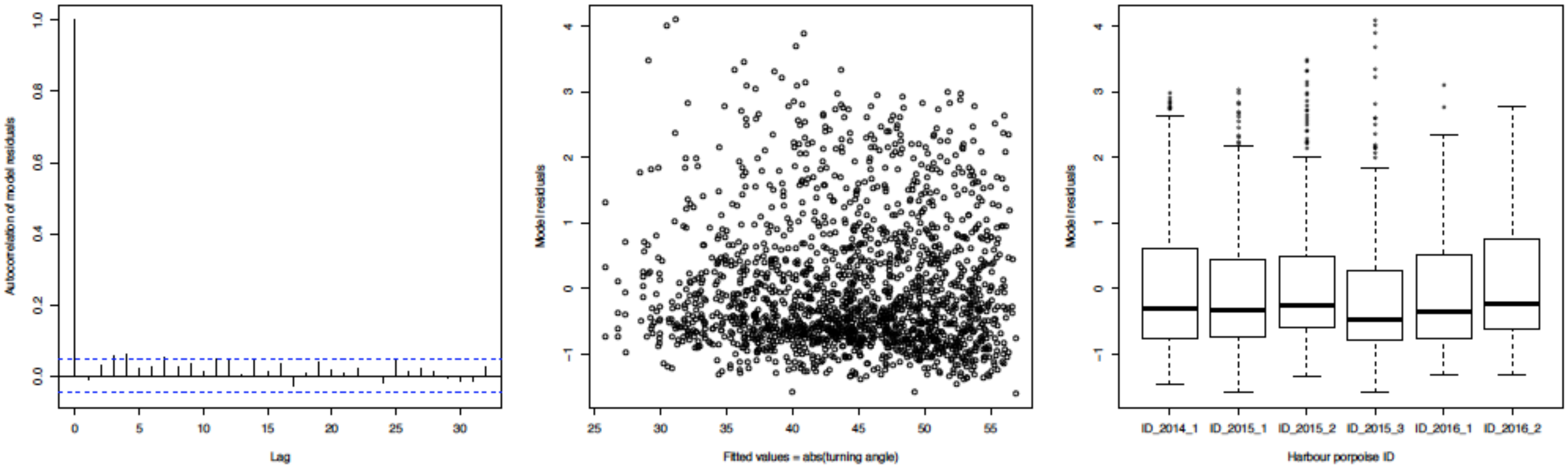

Fig. S6: Model validation plots to assess if the assumptions of linear regression for the absolute turning angle analysis are fulfilled. Left panel shows the autocorrelation of model residuals (independence of observations), the middle panel shows the fitted values versus model residuals (normality) and the right panel shows the spread of model residuals across porpoise individuals (homogeneity).

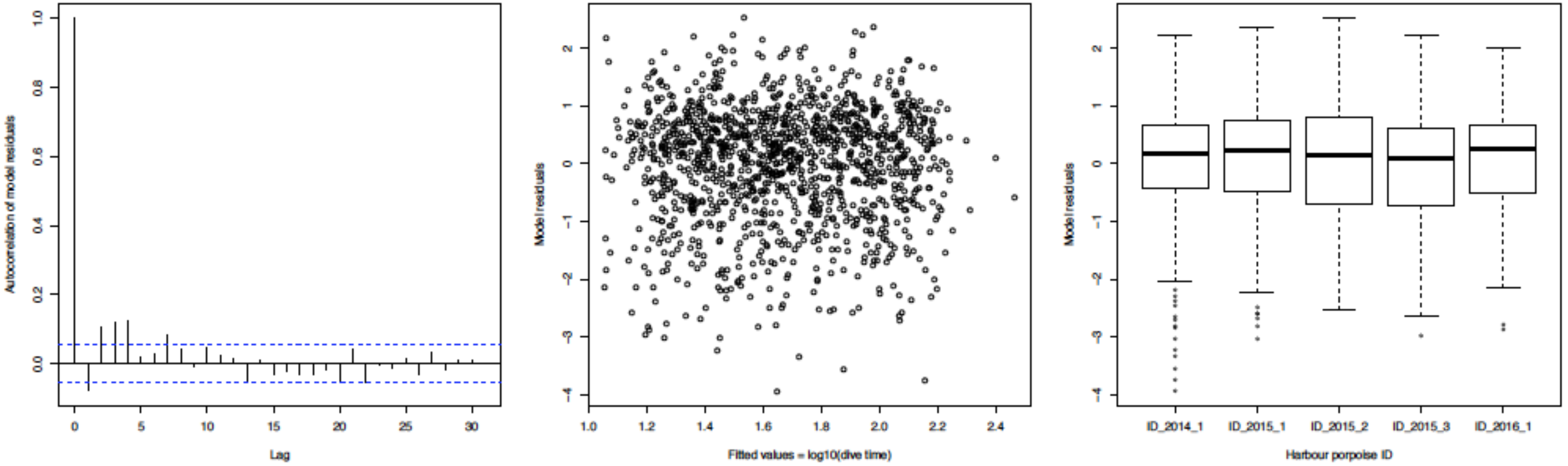

Fig. S7: Model validation plots to assess if the assumptions of linear regression for the dive duration analysis are fulfilled. Left panel shows the autocorrelation of model residuals (independence of observations), the middle panel shows the fitted values versus model residuals (normality) and the right panel shows the spread of model residuals across porpoise individuals (homogeneity).

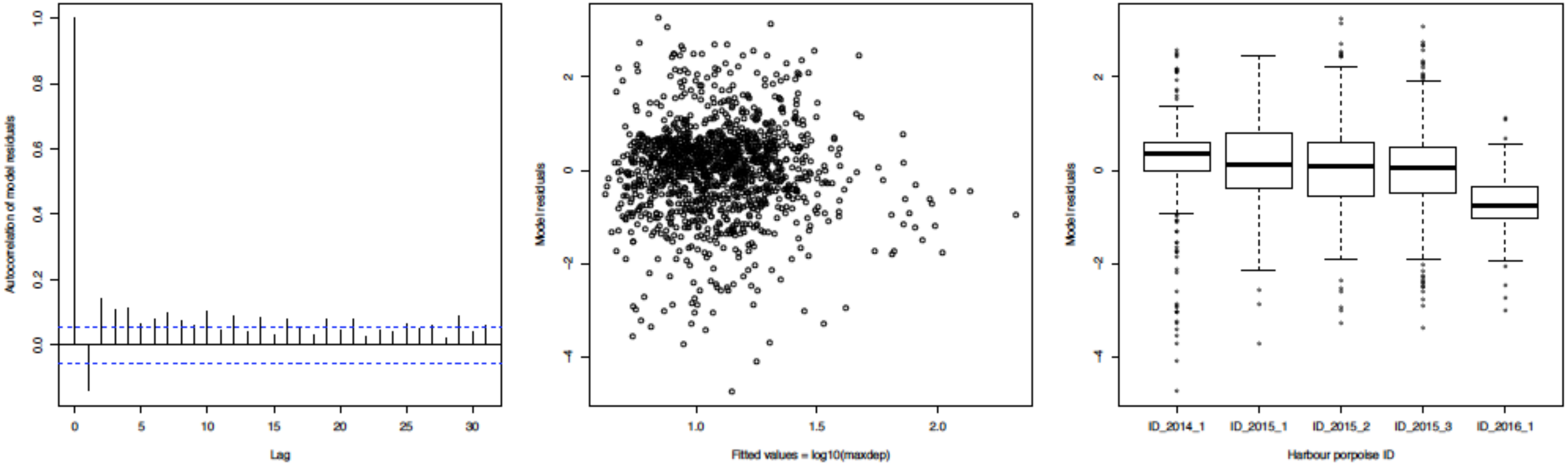

Fig. S8: Model validation plots to assess if the assumptions of linear regression for the dive depth analysis are fulfilled. Left panel shows the autocorrelation of model residuals (independence of observations), the middle panel shows the fitted values versus model residuals (normality) and the right panel shows the spread of model residuals across porpoise individuals (homogeneity).

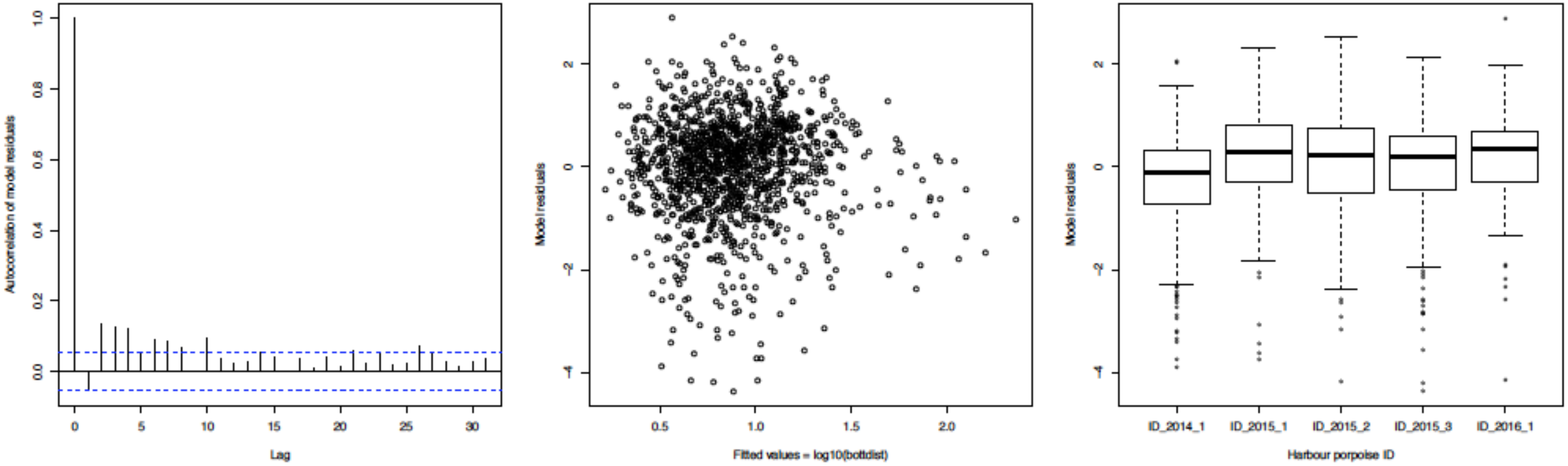

Fig. S9: Model validation plots to assess if the assumptions of linear regression for the dive wiggliness analysis are fulfilled. Left panel shows the autocorrelation of model residuals (independence of observations), the middle panel shows the fitted values versus model residuals (normality) and the right panel shows the spread of model residuals across porpoise individuals (homogeneity).

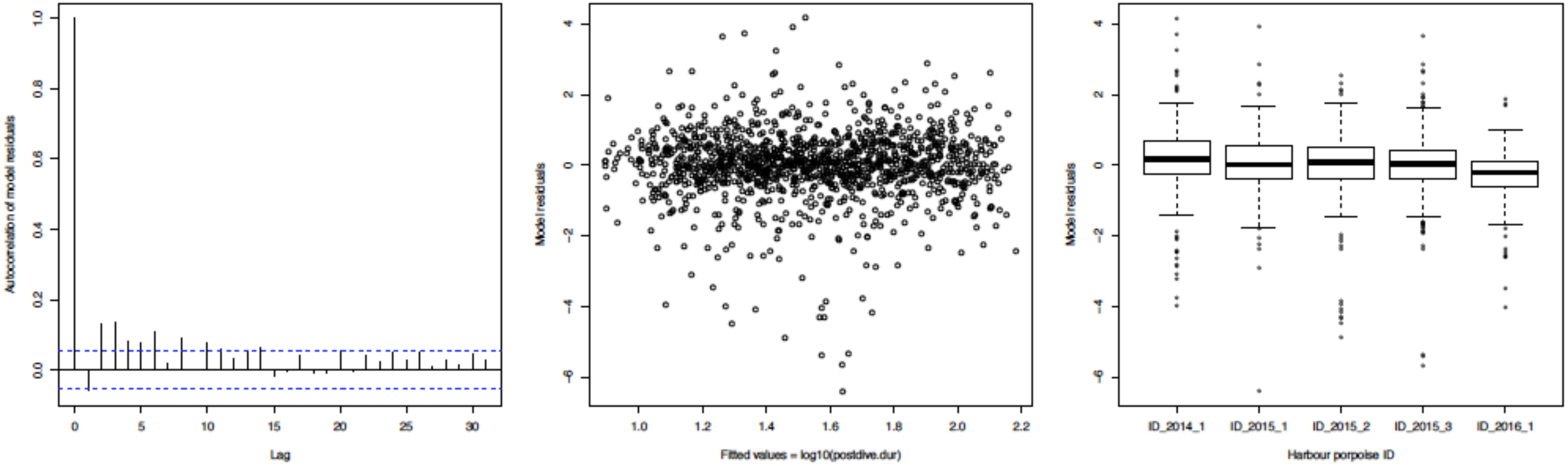

Fig. S10: Model validation plots to assess if the assumptions of linear regression for the post-dive duration analysis are fulfilled. Left panel shows the autocorrelation of model residuals (independence of observations), the middle panel shows the fitted values versus model residuals (normality) and the right panel shows the spread of model residuals across porpoise individuals (homogeneity).
